# Periodontal Disease Augments Cardiovascular Disease Risk Biomarkers in Rheumatoid Arthritis

**DOI:** 10.3390/biomedicines10030714

**Published:** 2022-03-19

**Authors:** Jeneen Panezai, Ambereen Ghaffar, Mohammad Altamash, Mikael Åberg, Thomas E. Van Dyke, Anders Larsson, Per-Erik Engström

**Affiliations:** 1 Section of Oral Health and Periodontology, Division of Oral Diseases, Department of Dental Medicine, Karolinska Institutet, 14104 Huddinge, Sweden; per-erik.engstrom@ki.se; 2 Department of Microbiology, Faculty of Life Sciences and Informatics, Balochistan University of Information Technology, Engineering and Management Sciences (BUITEMS), Quetta 87300, Pakistan; 3 Department of Applied Oral Sciences, The Forsyth Institute, Cambridge, MA 02142, USA; tvandyke@forsyth.org; 4 Rheumatology Clinic, Habib Medical Centre, Karachi 75950, Pakistan; drambereen@gmail.com; 5 Department of Periodontology, Altamash Institute of Dental Medicine, Karachi 75500, Pakistan; drmaltamash@gmail.com; 6 Clinical Chemistry, Department of Medical Sciences, Uppsala University, 75236 Uppsala, Sweden; mikael.aberg@medsci.uu.se (M.Å.); anders.larsson@akademiska.se (A.L.); 7 Department of Oral Medicine, Infection and Immunity, Harvard Faculty of Medicine, Boston, MA 02115, USA

**Keywords:** inflammation, proteins, proteomics, rheumatoid arthritis, periodontal disease, cardiovascular disease

## Abstract

Objectives: Periodontal disease (PD) and rheumatoid arthritis (RA) are known chronic conditions with sustained inflammation leading to osteolysis. Cardiovascular diseases (CVD) are frequent comorbidities that may arise from sustained inflammation associated with both PD and RA. In order to determine CVD risk, alterations at the molecular level need to be identified. The objective of this study, therefore, was to assess the relationship of CVD associated biomarkers in RA patients and how it is influenced by PD. Methods: The study consisted of patient (26 RA with PD, 21 RA without PD, 51 patients with PD only) and systemically and periodontally healthy control (*n* = 20) groups. Periodontal parameters bleeding on probing, probing pocket depth, and marginal bone loss were determined to characterize the patient groups. Proteomic analysis of 92 CVD-related protein biomarkers was performed using a multiplex proximity extension assay. Biomarkers were clustered using the search tool for retrieval of interacting genes (STRING) to determine protein–protein interaction (PPI) networks. Results: RA patients with PD had higher detection levels for 47% of the measured markers (ANGPT1, BOC, CCL17, CCL3, CD4, CD84, CTRC, FGF-21, FGF-23, GLO1, HAOX1, HB-EGF, hOSCAR, HSP 27, IL16, IL-17D, IL18, IL-27, IL6, LEP, LPL, MERTK, MMP12, MMP7, NEMO, PAPPA, PAR-1, PARP-1, PD-L2, PGF, PIgR, PRELP, RAGE, SCF, SLAMF7, SRC, THBS2, THPO, TNFRSF13B, TRAIL-R2, VEGFD, VSIG2, and XCL1) as compared to RA without PD. Furthermore, a strong biological network was identified amongst these proteins (clustering coefficient = 0.52, PPI enrichment *p*-value < 0.0001). Coefficients for protein clusters involved in CVD (0.59), metabolic (0.53), and skeletal (0.51) diseases were strongest in the PD group. Conclusion: Periodontal disease augments CVD-related biomarkers in RA through shared pathological clusters, concurrently enhancing metabolic and skeletal disease protein interactions, independent of autoimmune status.

## 1. Introduction

Chronic inflammation stems from persistent acute inflammation due to the failure to resolve the acute phase, often associated with the inability to remove the inducing agent or stimulus. Several diseases that acquire such chronicity due to a dysregulated immune response include atherosclerosis, type 2 diabetes, rheumatoid arthritis (RA), and periodontal disease (PD) [1].

Cardiovascular diseases (CVD) are the leading cause of global mortality with over 75% of cases in low- and middle-income countries [2]. CVD comprises a group of disorders that involve the heart muscle and blood vessels. The most common pathogenic pathway that leads to CVD is atherosclerosis [3]. Risk factors such as smoking, diabetes, hypertension, and obesity are transduced into atherosclerotic events via complex interactions between endothelial adhesion molecules and inflammatory cells including macrophages and T lymphocytes. The inflammatory response also has an autoimmune component as low-density lipoprotein (LDL) cholesterol, one of the retained lipids in atherosclerotic plaques, is antigenic resulting in production of high affinity antibodies [4].

PD is an independent risk factor for the development of CVD [5]. Systematic reviews have shown a consistent association between CVD and PD which may be partially attributed to shared risk factors and the dissemination of periodontal pathogens into the bloodstream or an increase in systemic inflammation [6].

RA is an autoimmune disease characterized by synovial inflammation and destruction due to immune mediated inflammation. This sustained inflammation in RA promotes cardiovascular pathology to such an extent that it remains the leading cause of mortality in RA patients [7]. The overall increased CVD risk in RA has been attributed less to traditional CVD risk factors and more to underlying autoimmunity and inflammatory burden [8].

The influence of PD and RA combined may increase the menacing effects of inflammation and raise an individual’s risk of developing CVD even further. This can be evaluated through exploration of emergent biomarkers involved in CVD initiation and pathology and, therefore, the aim of this study is to assess CVD related biomarkers in RA patients and how it is influenced by PD.

## 2. Materials and Methods

The study was performed at the Department of Periodontology, the Altamash Institute of Dental Medicine, between October 2012 and August 2017 in Karachi, Pakistan. Upon obtaining informed consent, a detailed questionnaire was used to acquire information pertaining to medical and dental history. The minimum sample size was calculated using the Epitools Epidemiological Calculators [9] with the assumptions of a power of 80% and a confidence interval (CI) of 95%. The parameters reported in the literature pertaining to South Asian population were used [10]: (1) frequency of 60% for PD among RA patients and (2) frequency of 28% for PD without RA.

### 2.1. Rheumatoid Arthritis Group

A total of 47 RA patients were recruited via consecutive sampling from the Department of Rheumatology at the Habib Medical Centre in Karachi, Pakistan. These patients were established RA cases diagnosed by a rheumatologist (AG) using current ACR/EULAR classification criteria [11]. All patients were receiving disease-modifying anti-rheumatic drugs (DMARDs), corticosteroids, non-steroidal anti-inflammatory drugs (NSAIDs), or a biologic DMARD (Rituximab) at the time of the examination. Based on their periodontal status, the patients were divided into two groups: RA with PD (*n* = 26) and RA without PD (*n* = 21).

### 2.2. Periodontal Disease Group 

A group of 51 participants diagnosed with PD, but exhibiting no signs of RA, gout, or osteoarthritis were also included. Individuals with a history of treatment for PD during the last six months and/or treatment with antibiotics in the last three months were excluded.

We used twenty controls for comparison and better characterization of the groups. All controls had clinically healthy periodontium and no systemic disease. Blood samples were drawn from all participants and prepared sera were stored at −80 °C until the time of analyses.

### 2.3. Periodontal Examination

Periodontal examination was carried out for all teeth except for the third molars by a single examiner (JP). The defining criteria for PD was probing pocket depth (PPD) of ≥5 mm (to the nearest millimeter) in at least three different sites using a periodontal probe (Hu-Friedy manufacturing, Chicago, IL, USA). Pockets measuring ≥5 mm were added to calculate the sum of deep pockets representing PD affected sites. Our group has designed continuous periodontal indices to gauge the severity of PD as a continuous variable rather than dichotomous. These parameters were used to assess the severity of PD:

- Bleeding on probing (BOP)
- ΣPPD Total
- ΣPPD Disease
- Adjusted PPD Total
- Adjusted PPD Diseased sites
- Σ Marginal bone loss (MBL)
- Adjusted ΣMBL

Details of the parameters and their measurements are described in our previous publications [12,13].

### 2.4. Anthropometric Measures

Body weight was measured to the nearest kg. Using non-stretchable measuring tape, height and waist were measured to the nearest cm. Waist was measured in the horizontal plane at the midpoint between the lowest rib and the iliac crest. Body mass index (BMI) was calculated from weight and height measurements (kg/m^2^). Anthropometric measurements were recorded for all four groups.

### 2.5. Glycated Hemoglobin (HbA1c)

Glycated hemoglobin levels were determined for all four groups after collecting four milliliters of whole blood into spray-coated EDTA tubes (lavender top, Becton, Dickinson, Franklin Lakes, NJ, USA). The samples were analyzed on the same day using the ion-exchange high-performance liquid chromatography system Bio-Rad D-10 Hemoglobin Testing System (Bio-Rad Laboratories, Hercules, CA, USA). The HbA1c values are standardized according to the National Glycohemoglobin Standardization Program (NGSP) system [14].

### 2.6. Proteomic Profiling

Serum samples were analyzed at the SciLifeLab Affinity Proteomics Uppsala, Uppsala University, (Uppsala, Sweden) using proximity extension assay (PEA) technology (Olink Proteomics, Uppsala, Sweden). Levels of 92 proteins from the Olink Target96 CVD II panel were measured (Supplementary File Table S1). The PEA technology utilize pairs of antibodies equipped with DNA reporter molecules [15]. When binding in close proximity to their correct targets, the antibody pairs give rise to new DNA amplicons each ID-barcoding their respective antigens. The amplicons are subsequently quantified using the Fluidigm BioMark™ HD real-time PCR platform (South San Francisco, CA, USA). For data analysis and quality control Olink NPX Manager Software was used and the inter-plate variability was adjusted by intensity normalization. The final protein values are expressed as Normalized Protein eXpression (NPX) values which are on a log2 scale and one unit higher NPX represents a doubling of the measured protein concentration. Data quality was controlled and normalized using an internal and an interpolate control. Assay validation data for all proteins from the panel are available (www.olink.com).

### 2.7. Protein–Protein Interaction (PPI) Network Analysis

The Protein–Protein Interactions (PPI) Network analysis was performed using the search tool for retrieval of interacting genes (STRING) (https://string-db.org, accessed on 21 February 2021). The STRING database interaction evidence is thematically grouped into ‘channels’ (such as text mining, co-expression, and lab experiments) and limited to *Homo sapiens*. An interaction score > 0.4 was applied to construct the PPI networks. STRING performed identifier mapping (test the proteins of each known pathway for any nonrandom skew within the user-provided input values, and report statistically significant pathways) and displayed a network with all of the mapped proteins and their interconnections [16]. In the networks, the nodes correspond to the proteins and the edges represent the interactions. STRING was employed to seek potential interactions among proteins. The clustering coefficient, where 0 represents the absence of connections and 1 a fully connected network, was calculated quantifying the abundance of connected nodes in a PPI network. PPI enrichment *p*-value is used to indicate that the nodes are not random and that the observed number of edges is significant.

### 2.8. Statistical Analyses

All analyses were performed test using GraphPad Prism version 9.0. for Windows (GraphPad Software, San Diego, CA, USA). Patient characteristics were analyzed using one-way ANOVA and Kruskal–Wallis tests depending on the normality of the data to identify group wise differences. Inter-group differences in biomarker distributions were analyzed using the Mann–Whitney U test. The relationship between each of the 92 protein biomarkers and periodontal parameters was assessed using Spearman correlation analyses. To control the false discovery rate (FDR), the Benjamini–Hochberg procedure was applied to adjust *p*-values from multiple testing [17]. The significance level was defined at *p* ≤ 0.05.

For exploration of biomarker patterns within disease groups, principal component analysis (PCA) was performed. PCA is a powerful exploratory model statistically used for data exploration and simplification. The technique is based on generating principal components (latent variable) from the original dataset. The relationship of the principal components to the samples is referred to as ‘scores’, and that to the variables is called ‘loadings’. A threshold of 0.5 was deemed significant for variable loadings.

## 3. Results

### 3.1. Characteristics of Study Groups

The characteristics for the disease groups (RA with PD, RA without PD, and PD only) and controls are shown in Table 1. There were no differences in age amongst the four groups. The number of females were higher in disease groups as compared to controls. The clinical status comprised self-reported hypertension and diabetes confirmed by medication and prescription. The frequency of both conditions were similar amongst the disease groups. Periodontal parameters, waist circumference and HbA1c differed significantly amongst the groups with the highest medians in PD patients. The median for HbA1c value in the PD group classifies them as pre-diabetes overall (Supplementary File Table S3).

### 3.2. Group-Wise Biomarker Distribution

**Figure 1:**
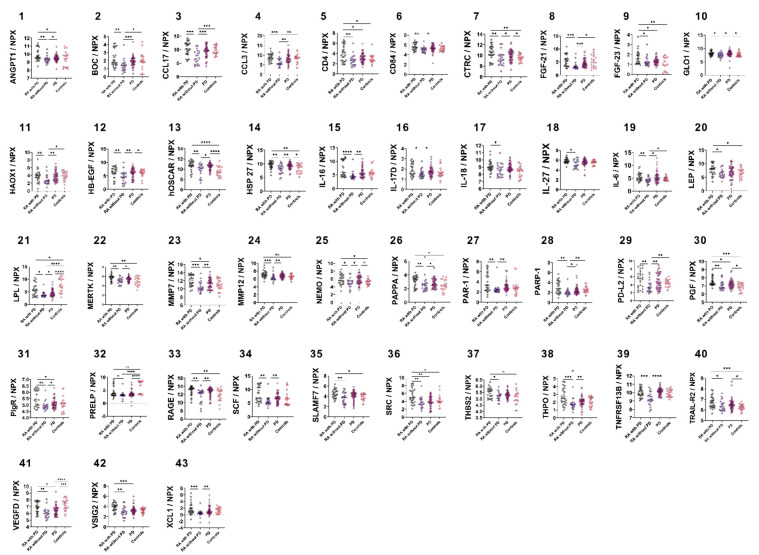
Group wise analyses for CVD-related biomarkers. Graphs 1–43 showing higher detection levels in RA with PD as compared to RA without PD. (1) ANGPT1, (2) BOC, (3) CCL17, (4) CCL3, (5) CD4, (6) CD84, (7) CTRC, (8) FGF-21, (9) FGF-23, (10) GLO1, (11) HAOX1, (12) HB-EGF, (13) hOSCAR (14) HSP 27, (15) IL-16, (16) IL-17D, (17) IL-18 (18) IL-27, (19) IL-6, (20) LEP, (21) LPL, (22) MERTK, (23) MMP7, (24), MMP12, (25) NEMO, (26) PAPPA, (27) PAR-1, (28) PARP-1, (29) PD-L2, (30) PGF, (31) PIgR, (32) PRELP, (33) RAGE, (34) SCF, (35) SLAMF7, (36) SRC, (37) THBS2, (38) THPO, (39) TNFRSF13B, (40) TRAIL-R2 (41) VEGFD, (42) VSIG2, and (43) XCL1. Data are presented as median with interquartile range. Group differences were calculated using Mann–Whitney U test. * *p* value ≤ 0.05, ** *p* value < 0.01, *** *p* value < 0.001, **** *p* value < 0.0001.

The distribution of 92 CVD biomarkers was assessed among the four groups. Two samples from the PD group were excluded due to unacceptable technical variations. Biomarkers with significantly increased levels in RA with PD groups as compared to RA without PD are shown in Figure 1. Higher NPX values were noted for 47% (43/92) of the markers which were: ANGPT1, BOC, CCL17, CCL3, CD4, CD84, CTRC, FGF-21, FGF-23, GLO1, HAOX1, HB-EGF, hOSCAR, HSP 27, IL16, IL-17D, IL18, IL-27, IL6, LEP, LPL, MERTK, MMP12, MMP7, NEMO, PAPPA, PAR-1, PARP-1, PD-L2, PGF, PIgR, PRELP, RAGE, SCF, SLAMF7, SRC, THBS2, THPO, TNFRSF13B, TRAIL-R2, VEGFD, VSIG2 and XCL1. For 32 of these biomarkers (BOC, CCL17, CCL3, CD84, CTRC, FGF-21, GLO1, HAOX1, HB-EGF, hOSCAR, HSP 27, IL-16, IL-17D, IL-18, IL-27, IL-6, LEP, LPL, MERTK, MMP12, NEMO, PAR-1, PARP-1, PD-L2, PRELP, RAGE, SCF, SLAMF7, THBS2, TNFRSF13B, TRAIL-R2, and XCL1) PD and RA with PD groups exhibited no differences (Supplementary File Table S2).

### 3.3. Correlation of CVD Biomarkers with Periodontal Parameters

The correlation between periodontal parameters and CVD-related biomarkers are shown in Table 2. The highest frequency of significant correlations was seen in the PD group for all parameters except for adjusted MBL. Anti-inflammatory marker IL-4RA was inversely related with three out of five indices for inflammation and pocketing. The Proto-oncogene tyrosine-protein kinase Src (SRC) was inversely correlated with four out of five indices for inflammation and pocketing. All correlations were direct amongst the RA with PD group, except for ACE-2. The most frequent and moderately strong correlations were noted with adjusted MBL. Least frequent correlations were noted in the RA without PD group. Dickkopf-related protein 1 (Dkk-1) and thrombospondin 2 (THBS2) were directly associated with Adjusted PPD Total. There was no overlap between the associated biomarkers amongst the three groups.

### 3.4. PCA

PCA was performed using standardized data and PC selection via parallel analysis. In the initial PCA output, selected component PC1 with loading structure >0.5 are shown for all disease groups (Table 3). The individual values show the correlation between the specific biomarker and the PC 1 for which the loading is calculated for. For RA with PD group, 64 biomarkers exceeded the 0.5 threshold of loading significance. Similarly, RA without PD had 65 biomarkers exceeding the threshold whereas PD group showed 55 biomarkers exceeding the threshold.

**Figure 2:**
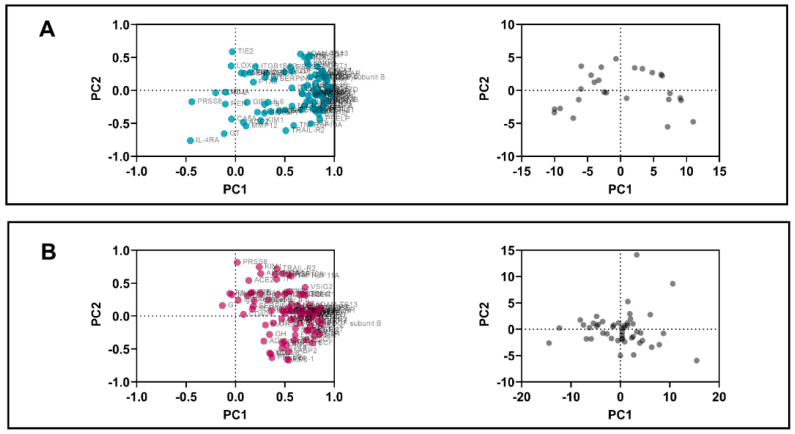

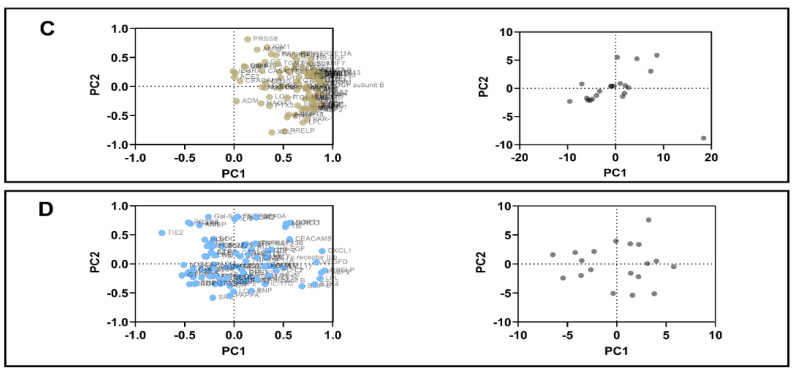
PCA analysis. The principal component analysis showing loadings (left side) and scores (right side) RA with PD (panel **A**), PD (panel **B**), RA without PD (panel **C**), and controls (panel **D**).

Visual representation of PC loadings plot (Figure 2) shows how the biomarkers are clustered closely together. In the disease groups, the majority of the biomarkers not only correlated strongly with each other but also with PC1 as most of the values were close to 1. The clustering pattern was more similar between the PD and RA without PD groups as some biomarkers showed stronger correlation with PC2. Using controls for reference, the loading plot showed weaker correlations between the biomarkers themselves and PC 1 and 2. The PC score plots reveal the variation in the dimensionality of the four groups.

### 3.5. Protein–Protein Interaction Network

**Figure 3:**
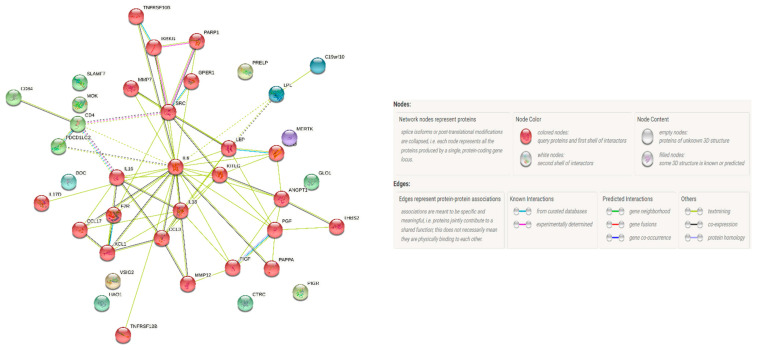
Protein–Protein interactions (PPI) showing networking of 43 CVD related biomarkers identified to be increased in RA with PD patients. The cluster shows frequent and strong interactions (represented by the same color of the nodes).

**Figure 4:**
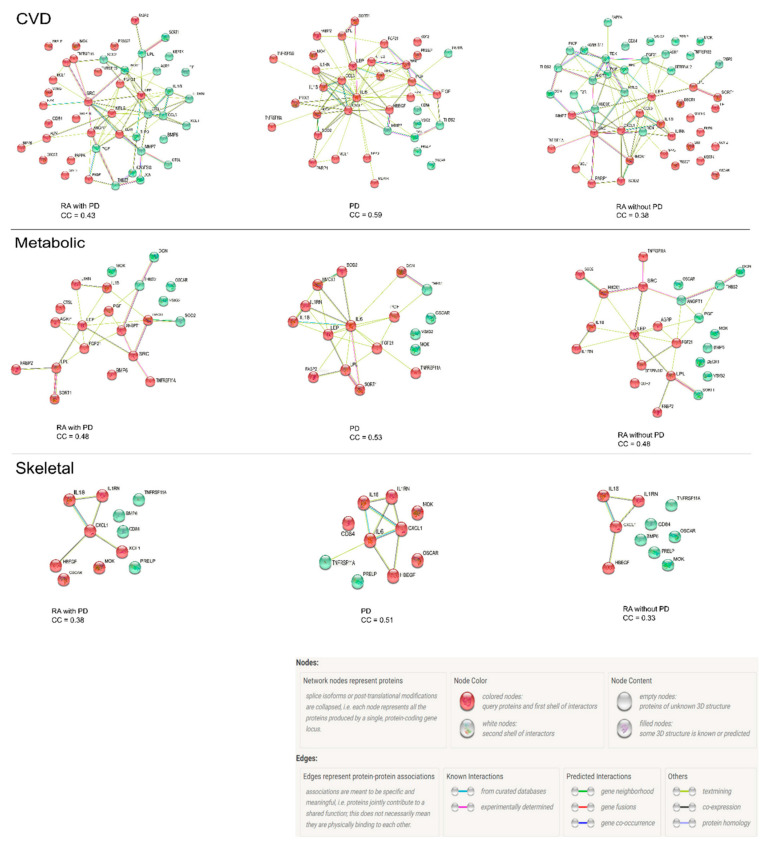
PC1 biomarkers and their protein network analysis according to disease area in RA with PD, PD, and RA without PD groups. The network nodes represent proteins with red colored nodes denoting first shell interactors and green color showing second shell of interactors. All cluster coefficients (CC) have a PPI enrichment value of <0.0001.

The Protein–Protein interaction (PPI) network analysis of 43 proteins discriminating RA with PD from RA without PD is shown in Figure 3. The potential interactions between ANGPT1, BOC, CCL17, CCL3, CD4, CD84, FGF-21, FGF-23, HB-EGF, hOSCAR, HSP 27, IL16, IL-17D, IL18, IL-27, IL6, LEP, LPL, MMP12, MMP7, NEMO, PAPPA, PAR-1, PARP-1, PD-L2, PGF, RAGE, SCF, SRC, THBS2, THPO, TNFRSF13B, TRAIL-R2, VEGFD, and XCL1 yielded a clustering coefficient of 0.52, with a PPI enrichment *p* value < 0.0001. Markers from the CVD panel that also play a significant role in metabolic and skeletal disease areas were identified from the PC1 results for each disease group based on bioinformatic databases, including UniProt, Human Protein Atlas, Gene Ontology (GO), and DisGeNET. PPI network analysis was performed for three disease areas per group. These results are shown in Figure 4. The clustering coefficient was strongest for the PD group in all three disease areas when compared to the RA groups. The metabolic disease proteins were identical in clustering strength in both RA groups, uninfluenced by PD status.

## 4. Discussion

In this report, we identified 43 markers with a strong interactive network in patients suffering from PD, with and without RA. The risk of CVD exists in both PD and RA through shared pathological clusters. Several markers also increase associated metabolic and skeletal disease risk, independent of autoimmune status. In order to prevent CVD related morbidity and mortality in chronic inflammatory conditions, it is crucial to identify and study CVD risk biomarkers in the early stages of inflammatory disease. Studying a vast array of biomarkers that are significant in CVD development is an advantage offered by protein profiling using proteomic techniques. The biological mechanisms can be better understood with identification of early stage biomarkers which predispose RA and PD independently or combined to risk of CVD. 

In our study, we examined an array of 92 biomarkers related to cardiovascular dysfunction and inflammation in RA patients with or without PD and PD patients alone. The disease groups showed a higher number of women of a relatively young age (<50 years). The gender dominance of women was expected since they are affected more by RA and seek dental care more frequently as compared to men [18]. In young women, being affected with RA is a risk for CVD [19]. RA female patients are 2.6-times more likely to develop CVD as compared to the general population. Our findings in relation to the age of the present cohort are, therefore, relevant.

An overall dysregulated level of HbA1c and increased waist circumference, a measure of central obesity, in PD patients has been confirmed previously as well [20]. Periodontal parameters were less severe in RA patients with PD and attributable to the use of disease modifying anti-rheumatic drugs (DMARDs) by the former group.

For direct comparisons, CVD biomarker distribution was assessed in all groups. Based on biological processes, the frequency of PD relevant biomarkers represented immune response (47%), cell adhesion (40%), intracellular mitogen-activated protein kinase (MAPK) signaling cascade (35%), inflammation (30%), catabolic process (23%), and proteolysis (19%). MAPKs are implicated as key regulators of inflammatory cytokines like IL-6 and TNF, thus transducing inflammation [21]. One of the contributing factors to CVD is endothelial dysfunction which is brought about by over expression of adhesion molecules due to inflammatory mediators [22].

The association of periodontal parameters with CVD biomarkers was also examined per disease group. In RA with PD, the associated biomarkers for periodontal pocketing spanned from enzymes (ACE-2) and membrane proteins (LOX-1) to plasma proteins (PTX3). The inverse relationship between ACE-2 levels and PPD Total scores reflect a pro-atherogenic state as ACE-2 levels have been detected in RA patients with a negative correlation with intima media thickness of carotid arteries [23]. Diseased probing sites correlated moderately with PTX3, also a pro-atherogenic inflammatory marker expressed by vascular endothelium known to modify angiogenesis and atherosclerotic lesion development [24]. Oxidized low density lipoproteins (ox-LDL) have been directly implicated in the pathogenesis of RA through signaling via the lectin-like ox-LDL receptor 1 (LOX-1) in the joint synovium [25]. LOX-1 activates downstream pathways that enhance atherosclerosis via endothelial dysfunction. LOX-1 is also expressed in platelets, where it enhances platelet activation and adhesion to endothelial cells [26]. Both LOX-1 and PTX3 associations with PPD Disease were moderately strong suggesting that PD contributes to a pro-atherosclerotic milieu in RA.

In PD patients only, three biomarkers (IL-4RA, SRC, MMP-12) conveyed a consistent pattern associated with deep pocketing. They reflect an unbalanced state with low anti-inflammatory IL-4RA levels confirming previous findings [27]. These findings corroborate a defect in the regulatory involvement of SRC and MMP-12 with phagocytosis and host defense mechanisms in PD patients. Low-MMP12 levels in periodontal tissues may be a risk factor underlying excessive pro-inflammatory IFN-γ macrophage activation in disease [28].

FGF-23, a bone-derived hormone, can also drive an increased production of pro-inflammatory cytokines [29]. Dkk-1 is known to play a pathophysiological role in bone erosion and joint remodeling in RA patients [30]. It negatively regulates the function of the Wnt pathway which is involved in the differentiation of osteoblasts. Thrombospondin 2 (THBS2) a matricellular protein, has been demonstrated as an endogenous regulator of angiogenesis and inflammation in the RA synovium [31].

High levels of LEP (leptin) associated with increased MBL in RA with PD patients further enforce previous findings of increased LEP levels found in dysfunctional immune phenotype including insulin resistance, inflammation, and disturbances in hemostatic factors [32]. TNSFR13B and its association with MBL reflects an increased B-cell proliferative and surviving capacity via its receptor BAFF (B-cell-activating factor). BAFF are up-regulated in RA synovial joints as well as early stages of PD [33,34]. IL-27′s enhancement of TNF-α mediated upregulation of adhesion molecules and pro-inflammatory IL-6 in blood monocytes of patients with acute myocardial infarction (MI) makes it high CVD risk associated [35]. The TGM2 levels in RA without PD correlate inversely with the total sum of MBL which aligns with previous findings that TGM2 correlates with RANKL production in human periodontal ligament cells as part of the inflammatory response in PD [36].

Protein biomarkers with high loadings (>0.5) on PC1 exceeded 50% of the total biomarkers analyzed in all disease groups. The biomarkers contributing to the greatest variance were similar in all three groups. Based on their disease–gene associations, these biomarkers are involved in vascular inflammation (HO-1, LPL, PAPPA, ADAMTS13, ADM, PGF, and GDF-2), hypertension, and arterial disease (PAPPA, ADAMTS13, ADM, and PGF). The underlying gene ontology represents upregulation of chemotaxis (XCL1 and CCL3), T helper 1 cytokines (SLAMF1, IL-18, XCL1, and IL-1ra), T helper 2 cytokines (XCL1), negative regulation of vasoconstriction (ADM and LEP), hematopoietic stem cell proliferation (THPO and ATXN) and increased bone loss (TNFSF11A and TF) [37].

The additional analyses of PPI networking for PC1 markers in CVD, metabolic and skeletal disease areas was performed as osseous and metabolic disturbances, especially insulin resistance, are highly frequent co-morbidities in both PD and RA [38,39,40,41]. The clustering coefficients displayed by PD group PC 1 biomarkers reflect a greater involvement of disease related proteins that make them a group with the highest risk for developing CVD, insulin resistance and skeletal diseases. The dampening of inflammatory circuits due to the use of NSAIDs and DMARDs in RA groups are to have some impact on the level of engagement amongst these proteins. Future studies are required to identify and validate markers of diagnostic and therapeutic relevance that may enhance the ‘treat-to-target’ strategy for RA and, hopefully, PD.

The limitations to our study pertain to the limited size of samples and the exploration of proteins which have been associated with cardiovascular diseases. Due to the exploratory nature of our study and the low prevalence of RA (~1%), we used a non-probability sampling method in which groups were not sex-matched. Despite these limitations, our findings have identified a direction for the exploration of other pathways in order to understand molecular alterations responsible for increased risk of CVD development in RA and PD.

## 5. Conclusions

We identified 43 markers with a strong interactive network in patients suffering from PD, with and without RA. In addition, several of these markers also increase associated metabolic and skeletal disease risk, independent of autoimmune status.

## Figures and Tables

**Table 1:** Characteristics for RA and PD (disease) groups and controls.

Characteristics	Disease Groups			Control	*p*-Value
	RA with PD (*n* = 26)	RA without PD (*n* = 21)	PD (*n* = 51)	(*n* = 20)	
Age (years) ^a^	48.5 (8.8)	43.1 (13.3)	47 (9.5)	43 (6.3)	0.11
Female sex, *n* (%) ^b^	21 (81)	20 (95)	33 (65)	8 (40)	<0.001
Clinical Status, *n* ^b^					
– Hypertension	9	7	8	-	0.14
– Diabetes	2	6	10	-	
BOP ^c^	23 (57)	43 (60)	77 (56)	15 (32)	<0.0001
PPD Total ^c^	301(81)	276 (86)	384 (113)	191 (24)	<0.0001
PPD Disease ^c^	107.5 (104)	0 (2.5)	229 (136)	0 (5)	<0.0001
Adjusted PPD Total ^c^	11.6 (2.9)	10.8 (2.8)	15.5 (4.2)	6.8 (1)	<0.0001
Adjusted PPD Disease ^c^	8 (4.3)	0 (0)	10.4 (4)	0 (0)	<0.0001
∑MBL ^c^	27.4 (10.8) ^d^	13.5 (12.9) ^e^	34.2 (15.4)	8.8 (17.5) ^f^	<0.0001
Adjusted ∑MBL ^c^	4.57 (1)	3.02 (0.9)	5.24 (2)	2.88 (0.8)	<0.0001
Body mass index (kg/m^2^) ^c^	24.2 (5)	24.1 (6.2)	25.2 (4)	23.9 (4.6)	0.35
Waist circumference (cm) ^c^	102 (30)	97 (23)	109 (19)	86 (17)	<0.0001
HbA1c % ^c^	5.0 (1)	5.0 (2)	5.7 (1.2)	4.5 (0.8)	<0.0001

BOP = bleeding on probing, PPD = probing pocket depth, MBL = marginal bone loss, HbA1c = glycated hemoglobin. ^a^ Differences in means were tested using one-way ANOVA test (testing overall difference among the three groups). ^b^ Differences in frequency were tested using χ² (chi-squared) test (testing overall difference among the three groups). ^c^ Differences in medians were tested using Kruskal–Wallis test (testing overall difference among the three groups). ^d^ Missing data (*n* = 5) was excluded in the analyses. ^e^ Missing data (*n* = 1) was excluded in the analyses. ^f^ Missing data (*n* = 1) was excluded in the analyses.

**Table 2:** Correlations of CVD risk biomarkers with periodontal pocketing and marginal bone loss.

	Periodontal Pocketing and Inflammation										Marginal Bone Loss			
	BOP		PPD Total		PPD Disease		Adj. PPD Total		Adj. PPD Disease		∑MBL		Adj. MBL	
	Analyte	r	Analyte	r	Analyte	r	Analyte	r	Analyte	r	Analyte	r	Analyte	r
RA with PD			ACE-2	−0.42	LOX-1	0.41			PTX3	0.44	ANGPT1 PGF	0.47 0.45	LEP TNFRSF13B IL-27	0.48 0.48 0.46
PD	CXCL1 SRC XCL1	−0.31 −0.32 −0.36	IL-4RA	−0.29	IL-4RA MERTK SRC	−0.37 −0.28 −0.29	IL-4RA MMP-12 SRC	−0.33 −0.31 −0.38	MMP-12 SRC	−0.34 −0.42	ADAM-TS13	−0.29		
RA without PD	FGF-23	−0.52					Dkk-1 THBS2	0.47 0.49			CD40-L TGM2	−0.47 −0.45		

Spearman rank correlation was used to identify correlations. All coefficients show biomarkers with adjusted *p*-values ≤ 0.05 after using the Benjamini–Hochberg procedure for multiple testing.

**Table 3:** PC loadings for disease groups.

	RA with PD			PD			RA without PD		
	Variable	PC1	PC2	Variable	PC1	PC2	Variable	PC1	PC2
	C22D4	**0.98**	−0.08	PDGF subunit B	**0.87**	−0.12	PDGF subunit B	**0.92**	0.02
	SCF	**0.96**	0.05	SOD2	**0.87**	−0.05	CD84	**0.90**	−0.14
	IL-17D	**0.95**	0.00	MMP7	**0.86**	0.12	SCF	**0.90**	−0.31
	PAR-1	**0.93**	−0.16	CD4	**0.85**	−0.28	BOC	**0.90**	−0.32
	BOC	**0.93**	−0.16	hOSCAR	**0.84**	0.08	CXCL1	**0.90**	0.11
	PIgR	**0.93**	−0.02	CCL17	**0.84**	−0.08	PD-L2	**0.89**	0.21
	VEGFD	**0.93**	0.20	IL16	**0.83**	−0.24	MERTK	**0.87**	0.22
	IL16	**0.93**	0.07	HB-EGF	**0.83**	0.05	VEGFD	**0.87**	0.06
	MMP7	**0.92**	0.15	CCL3	**0.83**	0.11	VSIG2	**0.87**	0.20
	SPON2	**0.91**	−0.14	PIgR	**0.80**	−0.30	MMP7	**0.86**	0.09
	PDGF subunit B	**0.91**	0.19	VEGFD	**0.80**	0.01	THBS2	**0.86**	−0.11
	THPO	**0.91**	−0.18	RAGE	**0.79**	0.07	BNP	**0.85**	−0.06
	CD84	**0.90**	0.21	SCF	**0.78**	−0.41	PIgR	**0.84**	−0.33
	hOSCAR	**0.90**	0.25	HSP 27	**0.78**	−0.21	PARP-1	**0.82**	−0.36
	LPL	**0.90**	−0.18	IL-17D	**0.77**	0.04	HO-1	**0.82**	0.16
	FABP2	**0.89**	−0.23	THBS2	**0.77**	−0.02	hOSCAR	**0.82**	0.27
	FGF-21	**0.89**	−0.30	CD84	**0.76**	−0.14	CD4	**0.82**	−0.26
	THBS2	**0.88**	−0.06	ADAM-TS13	**0.75**	0.17	CCL17	**0.82**	0.28
	CCL17	**0.88**	0.28	PD-L2	**0.75**	0.05	IL-17D	**0.82**	−0.20
	CXCL1	**0.88**	0.30	FGF-21	**0.74**	−0.06	DECR1	**0.81**	−0.17
	PARP-1	**0.88**	−0.10	HO-1	**0.73**	−0.22	FABP2	**0.81**	−0.41
	CTRC	**0.87**	0.26	Dkk-1	**0.72**	−0.22	GDF-2	**0.81**	0.15
	ANGPT1	**0.86**	−0.04	BOC	**0.72**	−0.36	RAGE	**0.80**	0.17
	PRELP	**0.85**	−0.44	PAPPA	**0.71**	0.03	SORT1	**0.80**	0.20
	MERTK	**0.85**	−0.06	VSIG2	**0.70**	0.44	PGF	**0.80**	0.28
	TM	**0.85**	−0.28	SORT1	**0.70**	0.32	ADAM-TS13	**0.80**	0.23
	CCL3	**0.84**	0.18	MERTK	**0.69**	0.07	CCL3	**0.80**	−0.23
	BMP-6	**0.84**	−0.36	GDF-2	**0.68**	0.33	FGF-21	**0.80**	−0.31
	SOD2	**0.83**	0.02	TNFRSF13B	**0.67**	0.11	HB-EGF	**0.78**	0.48
	SORT1	**0.83**	0.36	CXCL1	**0.66**	0.07	THPO	**0.78**	−0.39
	STK4	**0.81**	−0.07	GLO1	**0.66**	0.03	SLAMF7	**0.77**	0.39
	VSIG2	**0.81**	−0.04	NEMO	**0.66**	0.02	LEP	**0.77**	0.24
	SRC	**0.81**	0.10	CTRC	**0.66**	−0.02	Dkk-1	**0.77**	−0.22
	RAGE	**0.81**	0.24	SPON2	**0.65**	−0.11	TF	**0.76**	−0.01
	PGF	**0.81**	−0.31	CTSL1	**0.65**	0.32	AGRP	**0.76**	−0.14
	HO-1	**0.81**	−0.06	IL-1ra	**0.63**	0.16	IL-1ra	**0.76**	0.18
	FGF-23	**0.80**	−0.28	AGRP	**0.61**	−0.19	NEMO	**0.75**	−0.06
	IL18	**0.79**	−0.18	FGF-23	**0.60**	−0.29	TNFRSF13B	**0.74**	0.19
	Dkk-1	**0.79**	0.01	IL1RL2	**0.60**	0.08	PAR-1	**0.74**	−0.57
	AGRP	**0.78**	−0.27	THPO	**0.60**	−0.37	PSGL-1	**0.73**	−0.04
	XCL1	**0.76**	−0.50	DCN	**0.59**	−0.05	IL16	**0.73**	−0.39
	HB-EGF	**0.76**	**0.53**	IL-27	**0.57**	0.18	DCN	**0.73**	0.25
	HSP 27	**0.76**	0.20	TNFRSF11A	**0.56**	**0.61**	HSP 27	**0.72**	−0.01
	TNFRSF13B	**0.75**	−0.15	PTX3	**0.56**	0.08	PAPPA	**0.72**	−0.22
	PD-L2	**0.74**	0.50	BNP	**0.56**	−0.05	SOD2	**0.71**	−0.38
	NEMO	**0.74**	0.39	IL6	**0.55**	0.37	TNFRSF11A	**0.71**	**0.56**
	BNP	**0.74**	−0.08	TGM2	**0.54**	0.13	TM	**0.70**	0.32
	LEP	**0.73**	0.08	PGF	**0.54**	**0.63**	LPL	**0.70**	−0.62
	PAPPA	**0.73**	0.42	LPL	**0.54**	−0.67	FGF-23	**0.69**	−0.40
	IL-1ra	**0.72**	−0.17	PRSS27	**0.54**	0.08	GLO1	**0.66**	0.16
	TNFRSF11A	**0.70**	−0.24	FABP2	**0.53**	−0.53	SPON2	**0.65**	0.05
	DCN	**0.70**	**0.50**	PAR-1	**0.52**	−0.66	SRC	**0.64**	−0.33
	IL1RL2	**0.69**	0.04	TIE2	**0.52**	0.36	CTRC	**0.64**	0.10
	PSGL-1	**0.68**	−0.05	ANGPT1	**0.51**	−0.44	IL1RL2	**0.64**	−0.06
	TF	**0.67**	−0.05	IDUA	**0.50**	0.04	IL-27	**0.63**	0.47
	ADM	**0.66**	−0.30	IL18	**0.50**	0.33	PRSS27	**0.62**	0.38
	ADAM-TS13	**0.66**	**0.55**	SRC	0.49	−0.39	BMP-6	**0.62**	−0.49
	TNFRSF10A	**0.59**	−0.53	LEP	0.48	0.07	IL18	**0.61**	0.30
	MARCO	**0.57**	−0.24	TM	0.47	**0.64**	TIE2	**0.60**	**0.52**
	CTSL1	**0.57**	0.04	CEACAM8	0.47	0.09	SERPINA12	**0.57**	0.25
	GDF-2	**0.57**	0.29	STK4	0.47	−0.49	ANGPT1	**0.55**	−0.48
	PRSS27	**0.56**	0.35	PARP-1	0.47	−0.40	STK4	**0.54**	−0.53
	TRAIL-R2	**0.51**	−0.61	HAOX1	0.47	−0.04	ITGB1BP2	**0.54**	−0.20
	IL-27	**0.50**	−0.30	DECR1	0.43	0.33	REN	**0.53**	0.31
	GLO1	**0.50**	0.28	TRAIL-R2	0.42	**0.72**	PRELP	**0.51**	−0.77
	IgG Fc receptor II-b	0.44	−0.31	TF	0.42	**0.56**	IL6	0.49	0.01
	SERPINA12	0.39	0.17	TNFRSF10A	0.40	**0.63**	GH	0.47	0.15
	HAOX1	0.36	−0.32	ITGB1BP2	0.38	−0.10	GIF	0.46	−0.03
	IL6	0.33	−0.18	PRELP	0.37	−0.63	CTSL1	0.46	0.25
	DECR1	0.31	0.26	LOX-1	0.36	0.08	TGM2	0.44	0.39
	GH	0.30	0.20	BMP-6	0.36	−0.58	TNFRSF10A	0.42	**0.54**
	FS	0.29	−0.21	XCL1	0.34	−0.56	XCL1	0.38	−0.79
	SLAMF7	0.29	−0.35	GH	0.34	−0.28	TRAIL-R2	0.38	**0.56**
	KIM1	0.25	−0.46	Gal-9	0.33	0.24	PTX3	0.36	−0.33
	Gal-9	0.22	−0.33	MARCO	0.30	−0.14	LOX-1	0.36	−0.18
	ITGB1BP2	0.20	0.36	ADM	0.29	−0.38	KIM1	0.34	**0.68**
	PTX3	0.18	0.12	AMBP	0.26	**0.64**	CD40-L	0.32	0.07
	TGM2	0.15	0.27	CA5A	0.25	0.34	IgG Fc receptor II-b	0.32	−0.02
	GIF	0.12	−0.18	KIM1	0.24	0.74	GT	0.31	0.42
	MMP12	0.11	−0.54	PSGL-1	0.19	0.23	CA5A	0.29	0.26
	CEACAM8	0.09	0.25	SERPINA12	0.18	0.15	MARCO	0.29	−0.01
	ACE2	0.08	−0.49	SLAMF7	0.17	0.10	HAOX1	0.27	−0.29
	AMBP	0.06	0.26	GIF	0.17	0.36	IDUA	0.25	0.36
	TIE2	−0.03	**0.58**	REN	0.15	0.35	AMBP	0.23	**0.63**
	CA5A	−0.04	−0.44	ACE2	0.13	**0.54**	PRSS8	0.14	**0.81**
	LOX-1	−0.04	0.37	IL-4RA	0.12	0.31	MMP12	0.11	0.34
	IDUA	−0.10	−0.03	CD40-L	0.08	0.02	Gal-9	0.10	0.35
	REN	−0.10	−0.21	IgG Fc receptor II-b	0.03	0.24	CEACAM8	0.06	0.09
	GT	−0.11	−0.66	PRSS8	0.02	**0.81**	ADM	0.03	−0.25
	CD40-L	−0.20	−0.04	MMP12	−0.04	0.32	FS	0.01	0.23
	PRSS8	−0.44	−0.17	FS	−0.06	0.34	ACE2	0.00	0.16
	IL-4RA	−0.46	−0.76	GT	−0.13	0.16	IL-4RA	−0.01	0.26
**Proportion of variance**		**47.0%**	**8.9%**		**32.9%**	**11.6%**		**43.2%**	**11.3%**
**Cumulative proportion of variance**		**47.0%**	**55.9%**		**32.9%**	**44.5%**		**43.2%**	**54.5%**

PC = principal component. All loadings > 0.5 are in bold. The variance represented by two principal components in proportion and cumulatively are shown as percentages.

## Data Availability

All relevant data are provided as Supporting Information in Tables S1–S3.

## Supplementary Materials

The following supporting information can be downloaded at: https://www.mdpi.com/article/10.3390/biomedicines10030714/s1, Table S1, Olink Cardiovascular II panel; Table S2; Table S3, Clinical Parameters.
